# Persuasion with Limited Sight

**DOI:** 10.1007/s13164-018-0398-z

**Published:** 2018-05-23

**Authors:** Alex Lascarides, Markus Guhe

**Affiliations:** School of Informatics, University of Edinburgh, 10 Crichton Street, Edinburgh, EH8 9AB Scotland UK

**Keywords:** Human study of persuasion, Complex games, Manipulating preferences

## Abstract

Humans face many game problems that are too large for the whole game tree to be used in their deliberations about action, and very little is understood about how they cope in such scenarios. However, when a human player’s chosen strategy is conditioned on her limited perspective of how the game might progress (Degremont et al. 2016), then it should be possible to manipulate her into changing her planned move by mentioning a possible outcome of an alternative move. This paper demonstrates that human players can be manipulated this way: in the game *The Settlers of Catan*, where negotiation is only a small part of what one must do to win the game thereby generating uncertainty about which outcomes to the negotiation are good and which are bad, the likelihood that a player accepts a trade offer that deviates from their declared preferred strategy is higher if it is accompanied by a description of what that trade offer can lead to.

## Introduction

Models of dialogue have tended to focus on scenarios in which participants have no conflicts of interest: for instance, route descriptions (e.g. Anderson et al. (1991), Guhe and Bard (2008), and Tenbrink et al. (2010)) or shared problem solving (e.g. (Foster et al. 2008)). But dialogue with conflicts of interest also exist: for instance, negotiations over restricted resources (Cialdini 2009). Formal game theory is sometimes used to analyse such cases (e.g., Crawford and Joel Sobel (1982) and Rubinstein and Jacob Glazer (2006)). But these models assume that each participant has complete knowledge of their own payoffs in each possible end state of the dialogue (though not necessarily complete knowledge of their opponents’ payoffs). So they don’t handle cases where one is uncertain about which end states to the dialogue are beneficial, and which aren’t.

There are scenarios where this sort of uncertainty is very real, however. They arise when dialogue is only a part of what people must do to perform their task. For example, company mergers are highly complex: negotiation is a part of the activity, but not the only activity. When negotiating a divorce settlement, agreements about dividing the assets and child custody have long term impacts, some of which are (currently) unforeseeable; this creates uncertainty about which compromises in the negotiation constitute a good outcome. The board games Monopoly, Civilisation and The Settlers of Catan also involve a mix of linguistic negotiations and non-linguistic actions, such as buying property or attacking opponents with armies. In all of these examples, the game tree (of extensive form) that captures the state and action space may be finite, but the end states of the dialogue are *intermediate* states of the game, and the sheer size of the game tree makes it impossible for players—whether they’re artificial or human—to have complete and accurate knowledge of their own preferences over the dialogue’s possible outcomes. There are simply too many contingencies that can occur between the end of the dialogue and the end of the game to perform exact calculations.

Approximate inference of various kinds is deployed in practical systems tackling large game problems; for instance, Monte Carlo Tree Search (MCTS) (Browne et al. 2012). These methods rely on the agent having sufficient computational resources to construct several (and ideally, a representative set of) complete branches of the game tree, each one specifying an action sequence that connects the current state to an end state of the game. But human bounds on memory and processing mean that for very large games such as Settlers of Catan, Go and even Chess, many possible future contingencies are excluded entirely from the human player’s deliberations. And this complexity isn’t restricted to board games; they are a feature of real life (e.g., planning one’s career, deciding which house to buy, and so on). From the perspective of such a player, they have what (Degremont et al. 2016) call *limited sight* over the full game tree: there are (future) options that don’t figure at all in a player’s calculations on how to act now.

Assuming that human behaviour isn’t completely random, we need to model how humans decide their next move when they have limited sight, and perhaps know that they do. Frameworks for modelling agents with a limited (or flawed) perspective on the full game exist (e.g., Feinberg (2004) and Halpern and Rêgo (2013)). For instance, Degremont et al’s (2016) modal logic includes a modality $[!N]$ that restricts the agent’s capacity to foresee possible future outcomes: the formula ([!*N*]¬〈*a*〉¬*ϕ*) ∧〈*a*〉¬*ϕ*—which means that in the agent’s (restricted) perspective $\phi $ is always true after action *a* is performed, but (in actual fact), $\phi $ can be false after *a* is performed—is satisfiable.^1^ One can thus investigate how optimal policies within the scope of $[!N]$ diverge from those outside it.

These frameworks are useful for modelling artificial agents, but for *humans* they have two drawbacks. First, they don’t predict in what respects a human player’s perspective on the full (extensive form) game is limited, nor the factors that influence those limits. Presumably the limited sight is conditioned on the current game state (e.g., its distance to an end state, its branching factor, and/or the player’s prior experience of it), and on the player’s own properties (e.g., their expertise on the task, their attitude to risk, etc). As things stand, we lack a framework where the effects of these factors on which options get ignored in (human) reasoning can be investigated.

The second gap concerns applying standard solution concepts to *human* behaviour, regardless of whether they suffer from limited sight or not. Standard models of decision making choose actions that *maximise expected utility* (Savage 1954). But empirical evidence from behavioural economics shows that this Savagean approach doesn’t adequately model *human* decision making (Ariely 2008; Kahneman and Amos Tversky 1979). On the other hand, behavioural economics has so far focussed entirely on games where by design, they are sufficiently simple for the human to have ‘unlimited sight’ (see Fox et al. (2016) for an overview). How humans cope when deliberating over a limited portion of a massive game tree is largely unexplored.

Our current lack of knowledge of the phenomena is thus an obstacle to developing a formally precise but flexible framework for articulating and testing hypotheses concerning how humans attempt to solve complex game problems: the prior discussion shows that there are simply too many parameters one needs to consider. We must first *acquire data on human behaviour* in complex games, but in a principled and controlled way. One cannot obtain reliable evidence on the exact limits of a human player’s perspective on their options by simply demanding that they justify their current choice of action: the game is too complex for them to reliably report on their deliberations in an exhaustive manner. Instead, we need an experimental set up that probes the players’ limited sight more indirectly.

In this paper, we claim that a particular kind of *persuasion move* can serve this purpose. Persuasion is a dialogue move that a speaker performs with the intention of changing the recipient’s current plan. We describe an experimental set up in which one can estimate the likelihood that a player revises their current plan to an alternative plan that’s proposed to them, and that they do so on the basis that the proposer (also) mentions a future option that is made possible by the proposed plan. In effect, we measure the difference in likelihood that a player accepts a proposed plan that deviates from their current plan when a persuasion move of this kind is absent vs. when it is present. Note that the content of the persuasion move entails nothing at all about the *relative likelihood* amongst future states in the game (contra the persuasion moves studied by Rubinstein (2007) for instance). Rather, it *draws attention* to a future possible move. For the purposes of this paper, we view an utterance that draws attention to a (perhaps unforeseen) future possibility as one amongst many verbal contents that can be used to attempt to persuade someone to change their behaviour. This type of persuasive strategy is different from arguably the more typical type of persuasion strategy as studied by Rubinstein and many others, in which the utterance explicitly articulates arguments and/or information that to yields, via (commonsense) inference, conclusions about which strategy to prefer.

A player who had already considered but discounted the proposed plan in favour of their chosen plan, and whose calculations took into account the future possible option that the persuasion move draws attention to, is unlikely to be manipulated by the persuasion move: its content provides no new evidence to them. In this context, the only new evidence is the fact that the persuader implicates it’s a preferable option. A recipient may accept the move purely on these grounds—in effect, the recipient accepts the persuasion purely on the grounds of *who* said it rather than *what* was said, via an assumption that the persuader is *sincere* (i.e., she believes what she says) and *competent* (i.e., her beliefs about expected utilities in the game are true), or at least more competent than the recipient.

But one can minimise the chances that recipients accept the persuasion because of who said it, rather than what was said. First, one can design the experimental stimuli so that they provide no evidence at all about the persuader’s level of competence. And secondly, we can make the domain of persuasion a (complex) *win-lose* game: as Crawford and Joel Sobel (1982) show, Savagean interlocutors (defeasibly) infer that the speaker’s message isn’t credible in these circumstances. Accordingly, we can investigate, albeit indirectly, whether the *content* of the persuasion move (rather than who said it) prompts the recipient to revise their current plan. In other words, when recipients reject a proposed plan when it’s not accompanied by persuasion, but accept it when it is, the change of action is (probably) due to having their attention drawn to the future possible option mentioned in the persuasion. Or to put this another way, it is (probably) caused by the persuasion changing their (limited) perspective on how the game will unfold.

This paper presents a human study of the power of persuasion in the complex win-lose game *The Settlers of Catan* (or *Settlers* for short). Trade negotiations form only a small part of *Settlers*, and the game is too large for a human player to construct, let alone compare, complete sequences of actions, performed by themselves and their opponents, that connect the outcome of a trade to end states in the game. That is, the human *Settlers* player has limited sight. We test some hypotheses about how a persuasion move that simply mentions a future option of a (currently dispreferred) trade manipulates the player into accepting that trade.

Even though *Settlers* is a win-lose game, players have an incentive to negotiate and agree trades because the alternative ways of getting resources are more costly. But they’re not so costly that players will trade at any price; i.e., players can and do fail to agree trades. In this sense, negotiation in *Settlers* is similar to negotiations in commercial trade agreements, legal settlements, and so on. *Settlers*’ complexity also goes beyond the toy scenarios that are typically used to analyse negotiation (e.g., Binmore (1998), Brams (2003), and Rubinstein (2007)).

The structure of the paper is as follows. We start by describing the game *Settlers* (the domain of our study), and we highlight how we build and extend on prior work. We then present experiments in which we study when experienced human *Settlers* players accept a trade offer that deviates from their declared optimal strategy. In particular, we control whether the player’s attention is drawn to a future possible option of the proposed trade, or not. These experiments provide evidence for testing several hypotheses, the main one being that one can manipulate a human player’s strategy this way.

## The Settlers of Catan

We choose the board game *The Settlers of Catan* (or *Settlers*, catan.com (Teuber 1995)) for its complexity: it is multi-player, partially observable, non-deterministic and dynamic. Natural language negotiations form just a small part of the game. Its complexity means that players, whether human or artificial, can’t compute with complete certainty the actual preference function over the possible outcomes of a negotiation, either for oneself or one’s opponents (Cadilhac et al. 2015; Degremont et al. 2016).

**Fig. 1:**
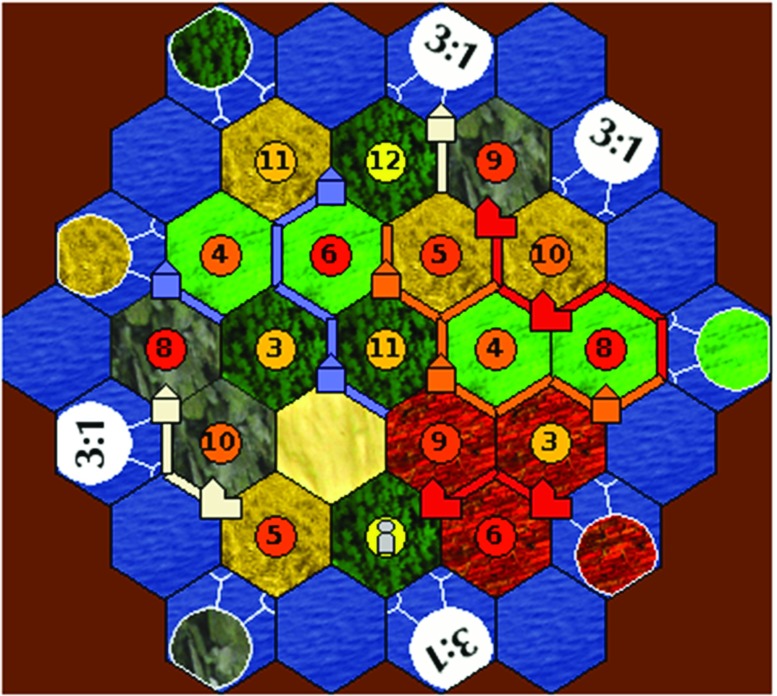
A game of *Settlers* in STACSettlers

*Settlers* is a win–lose board game for 2 to 4 players; you get Victory Points (VPs) by building settlements (1 VP) and cities (2 VPs) on a board of hexes (see Fig. 1), and the first player with 10 VPs wins. Each player acquires and uses resources (clay, ore, sheep, wheat, wood) to build roads, settlements and cities and to buy development cards, which have various functions (e.g., allowing you to move the robber).

Players can acquire resources by negotiating trades with other players and by the dice roll that starts each turn: if a player has a settlement (or city) on a hex whose number matches the dice roll, then she gets one (or two) of that hex’s resource. Dice rolls make the game non-deterministic.

Players can also gain (or lose) resources via *robbing*. When a player rolls a 7, she must move the *robber* (the grey piece in Fig. 1) onto another hex. This has three effects. First, the hex produces no resources via dice rolls until the robber is moved again. Second, the player who moved the robber can take one resource (at random) from an opponent who has a building on the hex. What is robbed is hidden to others, making the game states partially hidden. Third, any player with more than 7 resources must discard half of them; so there’s an incentive to use resources rather than hoard them. Deciding what resources to trade depends on what you want to build, e.g. a settlement needs 1 clay, 1 sheep, 1 wheat, 1 wood.

The size of the game *Settlers* is estimated in Table 1: *Depth* is the average number of executed actions (both dialogue and non-dialogue moves) from the start to the end of a game; *Branch* is the average number of legal actions in the encountered game states,^2^ with the figures drawn from two data sets. The Agent corpus consists of 1000 (simulated) *Settlers* games, where each (artificial) player is a version of the *Settlers* agent described in Guhe and Lascarides (2014b); the Human corpus consists of 60 games where each player is human (Afantenos et al. 2012).

**Table 1:** Estimated average game size, based on agent simulations and human play

Corpus	Branch	Depth
Human	63	152
Agent	69	234

The human corpus has several games with only 2–3 players, hence the smaller depth

## Related Work

### Related Work on Persuasion

Negotiation in game theory (e.g., Binmore (1998) and Brams (2003)) models when and how one suffers from the ‘winner’s curse’ (i.e., overpaying for an item, given the opponents’ preferences) and problems analogous to the prisoner’s dilemma (i.e., can one player trust the other to voluntarily cooperate during negotiation). Within game theory, standard negotiation models ascribe each player complete and static knowledge of her own potential payoffs over the negotiation’s possible outcomes (e.g., Binmore (1998)). So the associated models of persuasion focus only on the persuader manipulating her opponents’ *beliefs* about which outcomes are likely (e.g., Rubinstein (2007)) and the recipient predicting the *credibility* of such messages (e.g., Rubinstein and Jacob Glazer (2006)). The questions we address are complementary to this: when a player is uncertain about which end states to a negotiation will help her vs. hurt her, can a persuasion move that doesn’t express any information about likelihoods or beliefs manipulate her behaviour?

Studies on manipulating an opponent’s perception of which trades have a bigger payoff exist in argumentation theory (e.g., Amgoud and Vesic (2014)), but this work focusses on how the logical structures of two competing arguments determine which argument to accept and act on. We are interested in a problem that is independent of the logical structures of competing persuasion moves, for our focus is: if a player is uncertain about which trades help her vs. hurt her, then does drawing attention to a possible future outcome of a currently dispreferred trade enhance the likelihood that she chooses to execute that trade? Our persuasion moves don’t express any arguments as to why one should prefer that future option.

Persuasion has also been studied as an interpersonal phenomenon; e.g. Cialdini (2009) studies persuasive techniques in sales, using mechanisms like reciprocation, social proof or scarcity. Psychological theories that address persuasion include attribution theory and correspondent inference theory (Fiske and Taylor 1991; Heider 1958; Jones and Keith Davis 1965) as well as classic conditioning theories, going back to Pavlov’s famous experiments (Pavlov 1927). This paper doesn’t analyse these central aspects of persuasion, however. Rather, we focus only on providing data on human reactions to someone of unknown personality or type drawing attention to a future option of a (currently) dispreferred plan. Our hope is that this study can inform the development of a flexible framework for modelling how humans cope with complex tasks in which they have limited sight. We feel it is premature to decide what features that framework should have; the first step is to obtain empirical data, and this paper addresses that much more modest goal.

### Related Work on *Settlers*

Thomas (2003) built a symbolic agent that plays *Settlers* and an online *Settlers* gaming environment known as JSettlers. We adapted this gaming environment for our experiments. Machine learning approaches to modelling *Settlers* use Monte Carlo Tree Search (Dobre and Lascarides 2017; Szita et al. 2010; Roelofs 2012) and reinforcement learning (Pfeiffer 2003), and in addition to training on agent simulations they may also exploit a corpus of human play (Dobre and Lascarides 2015; Cuayáhuitl et al. 2015). But none of these machine learning approaches consider persuasion.

In trade negotiations, the persuading agent aims for either:

1. **More Trades for herself:** i.e., a desired trade she might not achieve otherwise (e.g., *If you accept this trade, you’ll get clay and be able to build a road*); or
2. **Fewer Opponent Trades:** i.e., she stops two opponents from trading with each other (e.g., *Don’t trade with him—he’s about to win!*)

Kraus and Lehmann (1995) propose hand-built symbolic strategies for performing both these kinds of persuasion moves within the complex game *Diplomacy*, but they aren’t evaluated in controlled and transparent ways. Agent simulations provide a practical means for evaluating how different persuasion and reaction strategies affect win rates. Indeed, we would argue that it is the *only* practical way of investigating this relationship: we found in previous work that testing the effect of a single factor on the win rate in *Settlers* requires a run of 10,000 games (Guhe and Lascarides 2012; 2014a; Guhe et al. 2013; Dobre and Lascarides 2015). The resources needed to collect this amount of data from humans is unfeasible. Furthermore, computer simulations are the only practical way of gaining the fine grained control one needs over the different factors that influence game play throughout the course of the game. Guhe and Lascarides (2014a) and Settle (2015) use game simulations to test the utility of various persuasion strategies against various response strategies in *Settlers*, validating particular hypotheses about when manipulating an opponent through persuasion enhances the win rate. In this paper, we complement this prior research by studying the extent to which *humans* can be manipulated via persuasion moves of the kind they used in these simulation studies with artificial agents.

## Negotiation and Persuasion in *Settlers*

In *Settlers*, there are many different kinds of persuasion moves that aim for More Trades, because there are many reasons for trading. Here is a small selection, drawn from attested data in a corpus of humans playing the game (Afantenos et al. 2012):

1. **Build**: ‘Give me 1 ore for 1 wheat and you can build a settlement, which you can’t build without the wheat.’
2. **Trade**: ‘Give me 1 ore for 1 wheat and only then will you have enough wheat to make a trade with your 3:1 port.’
3. **Block**: ‘If you give me 1 ore for 1 wheat, you can build a settlement that blocks Nick from the wheat port.’
4. **Buy Card & Get Largest Army**: ‘If you give me 1 ore for 1 wheat, you can buy a development card, and if you get a knight, you can get the Largest Army.’
5. **Rare Resource**: ‘If you give me 1 ore for 1 wheat, you will get a wheat resource to which you do not have access directly.’
6. **Too Many Resources**: ‘If you take this trade you will no longer have more than 7 resources and so you won’t have to lose half of them if someone rolls a 7’.
7. **Second Player Trade**: ‘If you give me 1 ore for 1 wheat, you can use the wheat to trade for Nick’s clay, so that you can build your road.’
8. **Always True**: ‘If you give me 1 ore for 1 wheat, I won’t rob you the next time I’m playing a knight card.’

We use a comparatively simple experimental set up for testing the effectiveness of persuasion (details are in Section 5). The main simplification is that we always pair a persuasion move with a trade offer that deviates from the participant’s declared preferences. So persuasion isn’t a response to previous persuasion attempts (contra the models of persuasion from argumentation theory (Amgoud and Vesic 2014)); nor do they refer to previous game actions or other dialogue contributions. Furthermore, we present a game state in isolation of the sequence of moves that generated it, and the participant can only give a yes/no answer to a trade offer which may, or may not, be accompanied by a persuasion move. Thus the human expert has no evidence of whether or not their opponent is trustworthy (as discussed in Section 1) and there is no basis for taking revenge on an opponent for their prior moves, because these are unknown.

A *necessary condition* for the persuasion to be credible is that its content is *consistent* with the recipient’s observable evidence—the content being both the proposition that the recommended move is possible if the recipient accepts the proposed trade, and its implied content that this move should be preferred.

We restrict the stimuli in our study to ones where these necessary conditions are satisfied. Since preferences over future options are always hidden, the implied content about what’s preferable is always consistent with observable evidence. But we also ensure in all our stimuli that the interlocutor’s observable portions of the game state are consistent with the proposition that the future action mentioned in the persuasion is made possible by the accompanying proposed trade.

## Human Reactions to Persuasion in Settlers

We now present experiments that test the power of persuasion in *Settlers*: does persuasion increase the chances that a human player will accept a trade offer that deviates from their declared preferred strategy? As we argued earlier, since our main motivation is to provide indirect evidence on how persuasion can manipulate a player’s perspective on how a game might unfold, we constrain the task in the following ways:

- The participants do the following when presented with a game state (which is sampled from game simulations to make them plausible): (i) they declare their preferred build action; and then (ii) on receiving a trade offer, they decide whether to accept or reject it. When the trade offer demands resources from the participant the she needs for her chosen build action (as declared in stage (i)), it is sometimes presented with an accompanying persuasion move that draws attention to a future option that’s made possible by the proposed trade, and sometimes not.

An alternative design would be to first present a trade offer to a participant, and then if she rejects it to repeat it with a persuasion move. But we rejected this design on the grounds that it is more complex and difficult to control, making it harder to collect enough data for a meaningful analysis: this is because if the rate at which participants *accept* the proposed trade offer without persuasion is high, then we would gain relatively few data points where they are presented with persuasion. Either way, this reduced setting doesn’t relate persuasion to winning the game. Instead, we measure the rate at which participants accept trade offers. This is the most important measure from our perspective: we want to investigate how manipulating a player’s limited sight on their future options also manipulates her into changing her actions (see Section 1 for motivation).

However, whilst the present human study doesn’t relate manipulation via persuasion to winning the game, Guhe and Lascarides (2014a) and Settle (2015) present *agent simulations* that show that successful persuasion will help you win, so long as you are persuading the opponent to execute trades that (on average) also help you win. More specifically, the extent to which a player’s persuasion strategy is successful correlates with their chances of winning the game, so long as the number of trades the player executes (also) correlates with winning. Our conjecture, therefore, is that any player that has a trading policy that is good enough to enhance her win rates increases her win rates still further against human opponents if she manipulates them into trading through persuasion.

Our main hypothesis for this experiment is Hypothesis 1:

*Hypothesis 1(absolute effectiveness).* People are more likely to accept atrade offer that deviates from their chosen plan when it is accompanied by apersuasion move than when it isn’t, where the persuasion move draws attention to aplan that is different from their chosen plan but made possible by the proposed trade offer.

The persuasion moves we use do not assert anything about the relative likelihood of the future option, nor anything about whether that future option should be preferred to its alternatives. Rather, the persuasion moves only draw attention to an alternative plan from the chosen plan. As we argued in Section 1, Hypothesis 1 being true is indirect evidence that the persuasion manipulates the recipient’s calculations of (expected) utilities amongst possible trades by expanding their limited sight on how the game might unfold: i.e., it can prompt a recalibration of what to prefer, or of one’s beliefs about what’s achievable, or both.

We also expect the *content* of the persuasion move to influence its effectiveness. In other words we would expect Hypothesis 2to be true:

*Hypothesis 2(relative effectiveness).* Different types of persuasion moves differ in their effectiveness: i.e., some types of persuasion enhance the likelihood of the accompanying trade offer being accepted more than others.

More specifically, what we mean by the term *effectiveness* in Hypothesis 2 is: persuasion of type $p1$ is more effective than $p2$ if and only if *in those game states where *$p1$*provides consistent information about what’s possible with the accompanying trade offer*, the difference in acceptance rates of that trade offer when $p1$ is present vs. absent is *larger* than the difference in acceptance rates when $p2$ is present vs. absent in those game states where $p2$ is consistent with the accompanying trade offer.

We test Hypothesis 2 using the following subset of persuasion types from the corpus study we discussed in Section 4 (see examples (1)–(5)):

- Build
- Trade
- Blocking another player (Blocking, for short)
- Buying a Development Card and getting the Largest Army badge (DC/LA)
- Getting an otherwise unobtainable resource from the trade (Rare Resource)

In fact, our experiments divide the Build move into four subtypes (see Section 5.1 for details), which vary on how many resources the player needs before the trade and after it in order to execute the build action. The rationale for not always reducing the needed resources to 0 to execute the recommended build plan is to counteract one’s incentive to use resources to build things whenever one can, rather than risk losing resources via a 7 roll.

It is natural to assume that an expert player is more confident in her choices than anovice player; if so, then one would expect experts to be less manipulable by persuasion than novices. On the other hand, that very same difference in confidence may mean that novices struggle more than experts to cope with the complexity in reasoning that’s required when *expanding* the size of the game tree that they are currently considering, and so feel unable to incorporate the future option that’s mentioned in the persuasion into any revised calculations of their next optimal move. Either way, there would be adifference in the extent to which experts vs. novices are manipulated:

*Hypothesis 3(experience).* The difference in likelihood of accepting atrade when accompanied by persuasion vs. not is different for expert players than for novice players.

### Design and Method

As mentioned earlier, we adopt a two-step approach for collecting data. When presented with a game state, participants first have to declare their preferred build action in that state; i.e., the build action they aim to execute next. This allows us to control whether the trade offer the participant then receives provides her with resources that she needs to achieve her declared goal, or takes those needed resources away. We thus have a means to compare the likelihood that a participant accepts a trade that *hinders* her from achieving her preferred build plan when it is accompanied with a persuasion move vs. when it isn’t accompanied by a persuasion move, thereby giving us a measure of the persuasion’s effectiveness at manipulating the participant into changing her actions.

**Fig. 2:**
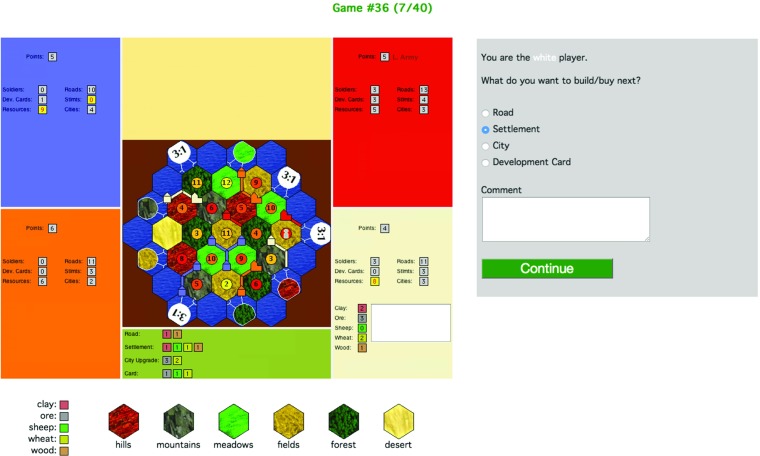
Stimulus phase 1 – eliciting the participant’s preferred build action

**Fig. 3:**
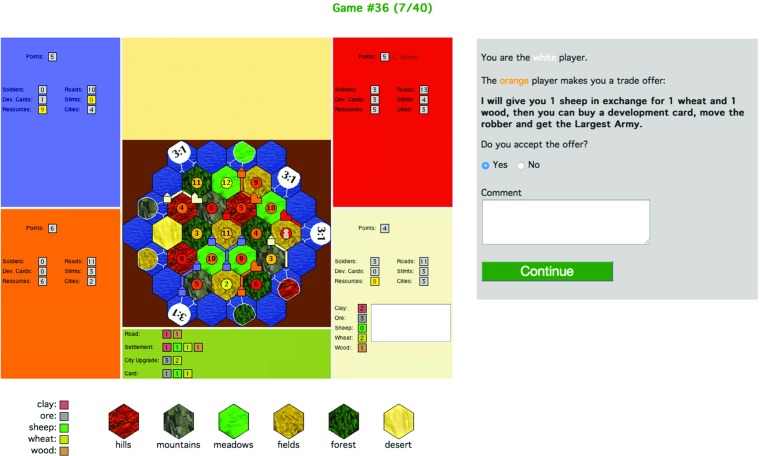
Stimulus phase 2 – responding to a trade offer (here: with persuasion move present)

Specifically, the experiment consists of the following steps:

- A game state is presented to a participant, and he or she is instructed to take on the role of one of the players. All the game states we used were such that there is at least one build piece (or development card) for which the participant needs either 0 or 1 resources to buy it. Furthermore, other than missing resources, nothing excludes any build action—e.g., there is a legal board position for building a settlement and development cards are on the draw stack.^3^
- The participant is asked which piece she is aiming to build next (road, settlement, city, development card); see Fig. 2.
- After the participant submits their choice, the screen is refreshed (the board continues to be shown), and she receives a trade offer from an opponent that allows her to build a different piece whilst at the same time taking a way a resource that she needs to build her chosen piece (target stimuli), or the trade offer enables her to build her chosen piece (filler stimuli). A target-stimuli trade offer is either accompanied by a persuasion move or not. The participant has to decide whether to accept the trade offer by choosing Yes or No; see Fig. 3.

Filler stimuli ensure that the participant sometimes receives trade offers that contribute to their chosen build plan; this avoids the risk of the participant noticing that all the trade offers they receive request resources that they need to achieve their chosen plan, which might compel them to lie about their choices. Target stimuli are sometimes accompanied by the mention of a build plan that the trade offer contributes to. For a filler stimulus, that build plan would be the one that’s already chosen by the participant. So unlike target stimuli, a ‘persuasion’ move for a filler stimulus doesn’t draw the participant’s attention towards an alternative build plan; nor does it attempt to change the participant’s actions to achieve a different goal, making it wrong to use the term *persuasion* to categorise such a move for filler stimuli. Nevertheless, one would ideally test whether mentioning the (chosen) build plan enhances the chances that the participant accepts the trade offer in a filler stimulus, but it’s not practical to do this within reasonable resources. Limits on our experimental resources meant we were restricted to 6 filler stimuli (and a similar number of target stimuli for each type of persuasion move). Given the very high rate at which the filler stimuli’s trade offers are accepted when the (chosen) build plan is *not* mentioned, one would need vastly more filler stimuli than we had, and certainly more than the number of target stimuli that we used, to test whether such a move in a filler stimulus enhances the participant’s chances of accepting the trade offer. Thus the *only* role that filler stimuli play in this experiment is to avoid participants receiving trade offers that always take from them resources that they need for executing their chosen plan.

The experiment has a 4 (build action) x 8 (persuasion type) x 2 (persuasion present vs. absent) design. As mentioned earlier, we split the Build move into 4 subtypes based on the number of resources that are missing for executing the player’s declared preferred build action before the trade offer is made, and the number of resources that are missing after the proposed trade is executed for performing the build action that is recommended in the persuasion. Before the trade, the missing resources could be 0 or $\geq $1 (there is guaranteed to be a build action with at most 1 resource missing, but the participant may choose a build action with more than 1 resource missing). The resources needed to fulfil the build action in the persuasion can be 0 or 1. So there are 4 subtypes of Build persuasion moves:

$$0 \to 0; 0 \to 1; \geq 1 \to 0; \text{ and }\geq 1 \to 1 $$

For example, if the participant possesses 2 clay, 3 wheat, 0 ore, 1 wood and 0 sheep and her declared build action is *settlement*, then this is a $\geq $1 case (she is missing 1 sheep to build a settlement). If she is offered 2 clay for an ore, with an accompanying persuasion that she build a city, then she needs one more ore to do this and so it is a $\geq $1 $\to $ 1 case.

The experiment software chose the type of persuasion to use (if at all) on the basis of the game state and the build action chosen by the participant. This software guarantees that the constraints we’ve specified on all persuasion moves in the experiment are satisfied: i.e., the persuasion is consistent with the participant’s observations of the game state; the build plan that’s recommended in the persuasion is distinct from the participant’s declared build plan; the trade gives a resource that the participant needs to execute the persuasion’s recommended build plan, leaving at most 1 resource still required to achieve it, and it requests a resource that’s needed for the participant’s chosen plan. If there were multiple persuasion arguments that satisfy these constraints, then which persuasion to present is chosen at random. On the other hand, stimuli where the participant chose a build action that would make all possible persuasion types conflict with the above constraints were ‘repurposed’ online as filler stimuli. For example, if the participant chose the *settlement* build action, then we cannot present a Block argument (‘… you can build a settlement and block the red player’), because the participant’s intended build action matches the recommended build action in the persuasion.

All (target and filler) trade offers are 2:1; that is, the participant must give away 2 resources in return for 1 resource. This is for two reasons: (a) the *Settlers* corpus attests many 2:1 trade offers (Afantenos et al. 2012); and (b) the strong ‘base’ incentive to accept 1:1 trades would undermine our capacity to evaluate whether persuasion has an effect. Thus trade offers that come with persuasion always started with the formulation: ‘I will give you an X in exchange for two Y, then $\dots $’ and continued (mutatis mutandis) as follows:^4^

- Build: ‘… you can build a city.’^5^
- Block: ‘… you can build a road and a settlement and block the red player.’
- Trade: ‘… you can trade with your wheat port and build a settlement.’
- Buy Development Card and Get Largest Army (BD/LA): ‘… you can buy a development card, move the robber and get the Largest Army.’
- Rare Resource (rare res): ‘… you get a wood, to which you don’t have access.’

We presented 8 target stimuli of the Build type and 6 of each of the other types. In addition, as well as the 6 filler stimuli (as motivated earlier), we also included 2 game states that act as ‘test cases’ in that they have a clear best next action: in these stimuli, both the participant and an opponent can build a settlement in the same location (because they both have a road of sufficient length leading to it), and the participant has the resources necessary to build a settlement. Building in such locations is very desirable in *Settlers*, because it not only gives 1 Victory Point but also blocks an opponent from building there. Thus each participant is presented with 40 stimuli in total.

#### Participants

were recruited via Edinburgh University’s Careers Service. We specified that participants would have to know the game prior to the experiment and have access to a web browser. There were no further restrictions, e.g. time of day, duration or operating system. Participants were paid £8 for their participation. 88 participants registered via a Web form. 50 completed the experiment. We excluded 1 participant from analysis, because it was obvious that he/she didn’t engage in the task.^6^ The analyses presented below are based on the remaining 49 participants: 26 male and 23 female. On average, they were 22.98 years old (median: 22.5; mode: 19). Participants indicated approximately how many games they’ve played during registration: $<$15 (N = 24), 15–30 (N = 9) or $>$30 (N = 16). All participants gave their informed consent during registration.

### Materials

We generated the stimuli for the experiment as follows.

#### Game States and Game Map

These were derived using a large-scale implementation of *Settlers* called STACSettlers (Guhe and Lascarides 2014b), which builds on the open-source implementation *JSettlers* (Thomas 2003).^7^ Like JSettlers, STACSettlers allows human players and computer agents to play each other in any combination, but STACSettlers features a number of enhancements. A relevant one here is its Replay Client (a tool for replaying games from log files)—we took screenshots of games that were played between four computer agents in simulations. All games were played on the standard game map from *The Settlers of Catan Game Rules and Almanack*.^8^

#### Resources

In each game state the participant needs 0 or 1 resource(s) to build at least one piece.

#### Turn

It is the participant’s turn.

#### Legal Options

In each game state, other than missing resources, the participant has a legal option for building a road, a settlement, a city and buying a development card. Specifically, in each stimulus the participant has:

- A legal place for building a settlement without having to build roads first, i.e., a location that’s at least 2 edges away from all settlements and cities, with two roads leading to it;
- A settlement that’s not yet upgraded to a city;
- At least one development card left in the deck;
- No option of making a bank or port trade without doing a trade first.

#### Needed Resources

The (target-stimuli) trade offer always removes the option of building the participant’s declared preferred build plan (but she may be able to build something else immediately after the trade).

#### ‘Unneeded’ Resources

The participant has at least 2 resources that she doesn’t need to execute the stimuli’s designated plan, which is the plan that requires 0 resources or 1 resource to be executed. This ensures that each stimulus has a 2:1 trade offer whose outcome is at least as close to making a build action possible as the game state before the trade.

#### Robber

The robber’s position is determined by the STACSettlers Replay Client, save for two constraints: (1) the robber is blocking a hex of the participant for the DC/LA argument; and (2) the robber is not blocking a hex of the participant in all other cases.

#### Current Victory Points for each Player

The VPs of each player is determined by the STACSettlers Replay Client. The target stimuli for Blocking had to satisfy a particular constraint on the players’ VPs, however. Blocking is distinct from the other persuasion moves in that it hurts one opponent to the (potential) benefit of all the others. Thus blocking can be viewed as forming a temporary alliance, over and above the temporary alliance of simply agreeing a trade. To mimimise the risk that the Blocking argument is accepted as a side effect of simply wanting to form a temporary alliance, we ensured that the opponent to be blocked had fewer VPs than the player offering the trade. In such contexts, it should be more important to the participant to hurt the player offering the trade than to hurt the one to be blocked.

#### Presentation

Each stimulus consisted of a STACSettlers screenshot showing the current game state, see Figs. 2 and 3. The following information is visible:

**Information about each opponent:** the number of Victory Points; Badges (Longest Road/Largest Army); the number of soldiers they’ve played; their number of unplayed development cards; total number of resources; Thus participants cannot form beliefs about the types of resources that opponents possess nor about their development cards—they do *not* have information about the game or chat history. (Consequently, the participant has less information than players in a real game.)
**Information about the participant’s hand:** As for opponents, plus the number of each of the 5 types of resources and a list of unplayed development cards.
**Legends:** Combinations of resources needed for building each type of piece (shown below the game board); colours used for the different resources (shown below the screenshot and in the participant’s hand panel); symbols used for the types of hexes (shown below the screenshot).

We modified the screenshots from the STACSettlers Replay Client in the following manner:

- The types (and number) of resources that the participant holds were changed to be suitable for the experimental case, in particular ensuring that the participant has the resources needed to make the designated persuasion argument consistent with the game state she would be in after executing a 2:1 trade. Further, we ensured that a bank/port trade isn’t possible without trading with an opponent first.
- Monopoly or Year of Plenty cards in the participant’s list of development cards were removed, and the number of development cards in the deck adjusted accordingly.
- All opponents have at least 1 resource and no more than 12, and the participant has no more than 8 (we don’t test the “Too Many Resources” persuasion argument, such as example (6)).
- For the DC/LA cases: (i) the participant’s number of soldiers was changed to match the number of soldiers of the opponent currently holding the badge; and (ii) one of the participant’s hexes is blocked by the robber.

For each stimulus, we verified that its designated persuasion move is consistent with it (see Section 5.3). See Appendix A for how we dealt with potential further factors.

### Procedure

Participants were directed to the website hosting the experiment via individual emails. The website was programmed specifically for this experiment, using a combination of scripts in PHP, JavaScript, jQuery and Perl as well as a MySQL database that contained information on the stimuli and recorded participant information and answers.

From the welcome screen, participants were directed to the registration page, to enter their name, email address, age, sex, English language proficiency, approximately how many games of *The Settlers of Catan* they had played (< 15, 15–30, $>$30) and some of their attitudes towards playing *Settlers*. They also gave their informed consent at this point.

After submitting the registration form, participants were shown a screen with the instructions (see Appendix B). Even though the participants’ comments were not our main goal, the instructions encouraged them because (i) it allowed us to determine whether participants were engaged in the task, and (ii) we could confirm that the stimuli did not contain any unintended properties that influenced the participants’ behaviour.

Stimuli were presented in a randomised order, generated by a PHP script.

Participants were first presented a game state, an example screenshot of which is shown in Fig. 2. They were instructed to take the perspective of a specified player. They were prompted with the question ‘What do you want to build/buy next?’. As answer, they had to make a forced choice between one of the four basic build actions (road, settlement, city, development card), and they could type in a free form comment (no restriction on length) and click on a button labelled *Continue*.

On clicking Continue, the chosen build action, the comment and the times and locations of mouse clicks after stimulus onset were recorded in the database. Depending on the stimulus type and the participant’s answer, the system presented a trade offer that sometimes was accompanied by a persuasion move—*which* trade offer and persuasion move were determined *after* participants declared their preferred build action so as to ensure that persuasion satisfies the constraints on its context of use that we described in Section 5.1. This was achieved as follows. The offering player, the giveable resource of the trade offer and the persuasion move were predefined for each target stimulus. By predefining these, we could perform consistency tests on their context of use before running the experiment rather than ‘online’: i.e., ensuring that the giveable resource is needed for the build action recommended in the persuasion, and the content of the persuasion move is consistent with the participant’s observed current game state. The two (receivable) resources that the trade offer demands in return for the giveable resource are computed ‘online’, so as to ensure that at least one of them is needed for the participant’s chosen action and consequently the target trade offer hinders the participant’s chosen build action, but helps them perform an alternative build action.

If the participant’s chosen build action is one where the predefined giveable resource and persuasion don’t satisfy the constraints on their context of use (e.g., the action recommended in the persuasion matches the participant’s chosen action), then the stimulus was converted online into a *filler* stimulus, and the trade offer presented to the participant was generated online to be one that gives a resource that’s needed for her chosen build action without taking needed resources away (so in contrast to target trade offers, it doesn’t hinder the participant’s chosen build plan). Otherwise, the *target* trade offer (computed as we’ve described) is presented, and in half the cases, chosen randomly, the (pre-defined) persuasion move is presented as well.

With the trade offer presented (either with or without an accompanying persuasion move), participants were asked whether they wanted to accept the trade offer or reject it.

All trade offers were stored in the database together with the participants’ response. After clicking the Continue button, the next stimulus was presented. After the last stimulus, participants answered a short questionnaire that we used as a ‘sanity-cheque’ on the experimental design and to ensure participants were engaged in the task. Participants needed 75 minutes on average to complete the experiment, including registration.

### Results

*Hypothesis 1(absolute effectiveness).* People are more likely to accept atrade offer that deviates from their chosen plan when it is accompanied by apersuasion move than when it isn’t, where the persuasion move draws attention to aplan that is different from their chosen one but made possible by the proposed trade offer.

Table 2 gives an overview of the results. The average acceptance rate for trade offers increases from 0.219 without persuasion to 0.332 with (over all persuasion types). A binomial test shows that this increased likelihood is statistically significant (p $<$ 0.001).^9^ Thus Hypothesis 1 is true, according to our experimental evidence.

**Table 2:** Overview of results (N = 49)

type	persuasion used				no persuasion				ΣΣ	binom
	yes	no	${\Sigma }$	yes/Σ	yes	no	${\Sigma }$	yes/Σ		$(p<0.05)$
0$\rightarrow $0	12	68	80	0.150	10	61	71	0.141	151	no
0$\rightarrow $1	5	20	25	0.200	3	14	17	0.176	42	no
1$\rightarrow $1	18	51	69	0.261	10	44	54	0.185	123	p = 0.075
1$\rightarrow $0	15	30	45	0.333	7	31	38	0.184	83	yes
x$\rightarrow $y	50	169	219	0.228	30	150	180	0.167	399	yes
x$\rightarrow $x	30	119	149	0.201	20	105	125	0.160	274	no
block	53	54	107	0.495	48	78	126	0.381	233	yes
trade	26	80	106	0.245	8	114	122	0.066	228	yes
DC/LA	57	53	110	0.518	31	112	143	0.217	253	yes
rare res	84	61	145	0.579	59	96	155	0.381	300	yes
Σ	350	705	1055	0.322	226	805	1031	0.219	1413	yes
test case					67	33	100	0.670	100	
filler					348	139	487	0.715	487	
ΣΣ									2000	

*yes* columns are the number of (target) trade offers that were accepted, *no* columns are the number that were rejected, and $yes /\sum $ is the proportion of trade offers that are accepted. These are separated according to whether persuasion was attempted *(persuasion used)* or not *(no persuasion)* The final column specifies whether the trade offer with persuasion is significantly more likely to be accepted than that same offer without persuasion. As well as giving the results separately for the 4 subtypes of Build arguments x $\rightarrow $ y is the sum of all Build cases and x $\rightarrow $ x the sum of the 0 $\rightarrow $ 0 and 1 $\rightarrow $ 1

However, whilst the aggregate of all Build arguments (x→y) has a significant effect (*p* = 0.01), there was no significant effect for 0→y cases; i.e., for those cases where the participant could immediately execute her preferred build action without trading. However, for all four subtypes of Build move, the absolute numbers of successful trade offers are higher when the persuasion move was used than when it wasn’t; i.e., the difference is in the same direction for all four subtypes. Further, we collected substantially fewer data for each of these four subtypes than for the other types of persuasion move. This, together with the sum of all Build moves having significant effects, suggests that the lack of effect is down to not collecting enough data.

As we mentioned earlier, no filler stimuli were accompanied with amention of abuild plan (which in this case would have been the participant’s chosen plan). The high acceptance rate without such amove confirmed our prior suspicion that the number of filler stimuli we had available prohibits the possibility of significant effects. In fact, in all filler stimuli where trading is necessary to execute the chosen plan, the participant accepted the trade offer without apersuasion move. In filler stimuli where the participant didn’t need to trade to execute her chosen plan, accepting the trade offer isn’t necessary and would result in possessing fewer resources because the trade is 2:1.

*Hypothesis 2(relative effectiveness).* Different types of persuasion argument differ in their effectiveness to change people’s behaviour.

Table 3 shows our measure for the *persuasive power* of the different persuasion types (in the final column), viz. the increase in accepted trade offers (the difference of *yes* answers between the persuasion vs. no persuasion cases) relative to the sum of accepted trade offers (the sum of all *yes* answers), as defined in equation (1):

1 $$ \mathrm{\frac{\mathit{yes}(persuasion) - \mathit{yes}(no\ persuasion)} {\mathit{yes}(persuasion) + \mathit{yes}(no\ persuasion)}} $$

Table 4 shows the pairwise comparison of the persuasive power, using tests of proportion (the *prop.test* function in the R statistical package).^10^ Whilst there are no significant differences between Build and Rare Resources or between Build and DC/LA, considering the overall results, this gives the following ranking in increasing order of persuasive power: Blocking (0.05); Rare Resources (0.175); Build (0.250); DC/LA (0.295); Trade (0.529).

**Table 3:** Relative effectiveness of types of persuasion arguments

type	persuasion used			no persuasion			diff[pers	Σ(yes)	pers.
	yes	${\Sigma }$(pers)	yes/Σ	yes	${\Sigma }$(no pers)	yes/Σ	–no pers]		power
block	53	107	0.495	48	126	0.381	5	101	0.050
rare res	84	145	0.579	59	155	0.381	25	143	0.175
x$\rightarrow $y	50	219	0.228	30	180	0.167	20	80	0.250
DC/LA	57	110	0.518	31	143	0.217	26	88	0.295
trade	26	106	0.245	8	122	0.066	18	34	0.529

**Table 4:** Effects of relative effectiveness

	block	rare res	x$\rightarrow $y	DC/LA	trade
block	—	yes	yes	yes	yes
rare res		—	p = 0.429	yes	yes
x$\rightarrow $y			—	p = 0.627	yes
trade					—

One might suspect that this ranking in persuasive power is predicted by whether the persuasion move results in an immediate gain in Victory Points (VPs), or not. It would be natural for a player who is unsure of what moves enhance the chances of eventually winning to favour those moves that afford a clear immediate benefit. However, whilst there is some positive evidence that the binary feature of immediately gaining VPs (or not) influences the persuasive ranking in *Settlers*, it cannot be the sole influence. Build and Trade both result in adding (permanently) 1 VP to your score; DC/LA adds 2 VPs to your score (which you will lose if an opponent subsequently plays more soldiers than you). Blocking and Rare Resource don’t result in an immediate gain in VPs. So this (binary) feature of gaining VPs immediately (or not) would explain why Build, DC/LA and Trade are more potent than Blocking and Rare Resource, but it doesn’t explain why Rare Resource is more potent than Blocking, nor why Trade is more potent than DC/LA or Building.

There are many negotiating games where immediate gains are possible in addition to long term rewards, and (like *Settlers*) it is not always clear that an immediate gain is worth it. For example, imagine you are the CEO of a company and you’re engaged in a (complex) negotiation with a multi-national corporation who wants to buy it. You might see your stock price increase significantly when you resolve a particular issue in the buy out—e.g., when you agree on where the new head quarters is to be located. So it may be tempting to come to such an agreement, but not so tempting that you will agree to any location: resolving the issue in a way that is detrimental to the final deal may not be worth the immediate (and temporary) increase in stock price. Thus one would expect the extent to which immediate benefits influence persuasive power to vary from one negotiating game to another, and (as in *Settlers*) to be one of several factors that influence persuasive power.

The second factor that we suspect is influencing the observed ranking in persuasive power—and in particular the ranking of Rare Resource to Blocking, and Trade to all the others—is the *visual saliance* of those aspects of the game state that determine whether the build plan that’s mentioned in the persuasion move is possible, or not. For instance, whether or not you can Block an opponent is dependent on the presence of a specific configuration of roads and settlements on the game board. This configuration is arguably highly visually salient: it’s depicted in a continuous portion of the game board, in a central part of the screen (see Fig. 2), making it easy to spot if Blocking is feasible. So there is every reason to expect that a participant notices the opportunity to Block in the relevant target stimuli, putting that (future) option within her limited sight of how the game might unfold when in the first stage of the experiment—i.e., when she is asked to choose her preferred build plan. In contrast, to identify a Rare Resource relies on noticing much less visually salient features of the state: one must notice a *lack* of settlements on all hexes of that resource, and the absence of a settlement in several positions on the board is plausibly harder to spot than blocking.

Rare Resource (and Blocking) don’t depend on numeric calculations, however, whilst Building, DC/LA and Trade do, and these numeric calculations aren’t depicted at all on the screen. Further, these numeric calculations depend only on the needed resources you currently possess, which are shown in a small area towards one corner of the screen (see Fig. 2). Trade requires you to identify and reason with both the needed resources and your available unneeded resources for trading with the bank (or port); so the visual information that makes Trade feasible is visually more distributed than for the Build or DC/LA options.

Overall, then, the ranking in persuasive power shown in Tables 3 and 4 correlates roughly with a combination of (a) whether the plan proposed by the persuasion results in an immediate benefit, or not; and (b) the extent to which the feasibility of the plan proposed by the persuasion is visually salient (including whether the option requires numeric calculations, which aren’t visualised at all)—the above discussion shows that this latter factor would explain why Trade is more potent than DC/LA and Build, and also why Rare Resource is more potent than Blocking.

Our experiments don’t test whether an option’s visual salience correlates with it being within the player’s deliberations when choosing her preferred plan. As we said earlier, it’s not possible to obtain reliable data, even through self-reflection, about what’s within a player’s limited sight of the game. It’s also very difficult to quantify the degree to which a given aspect of the game state is visually salient. However, the correlation between the player’s limited sight and visual salience is a highly plausible assumption, and if correct then the ranking in persuasive power we’ve observed suggests that the potency of a persuasion move is (negatively) affected by the likelihood that the participant already considered it when choosing her preferred build plan.

We have only suggestive evidence that immediate benefits and visual salience affect the potency of a persuasion move, however. To gain conclusive evidence, we would need to conduct experiments where we control the way the game state is visually rendered on the screen: e.g., varying the size and position of the game board relative to that of the participant’s resources, and varying whether the quantity and type of resource that the participant still needs to build various pieces are shown on screen or (as now) not. Manipulating vision and perception to test its impact on persuasion is a matter of future work, however, and beyond the scope of this paper. Nevertheless, if visual salience is an influencing factor in *Settlers*, then it is highly likely that other complex board games like Chess, Go, Monopoly, Civilisation and Diplomacy, share this property.

Note that Blocking, the only persuasion type we used where accepting the move puts the participant in atemporally alliance (over and above that of agreeing atrade) with other players, was the least effective persuasion type. This fact, together with the feature in our Blocking stimuli that the player offering the trade has more VPs than the player to be blocked, suggests our experimental design successfully mitigated against temporary alliances being afactor in the participant’s decisions on trading.

*Hypothesis 3(experience).* The likelihood that expert players change their minds when apersuasion move is given is different from that for novice players.

Table 5 shows the results by experience according to their self-assessment during registration. To streamline the presentation, we only distinguish between two categories: ‘novices’ (with fewer than 15 games) and ‘experts’ (with 15 or more games). Novices are almost twice as likely as experts to accept a trade offer (p $\ll $ 0.01), but persuasion leads to a significantly higher increase in accepted trade offers for experts (0.274 from 0.138) than for novices (0.433 from 0.312; p $\ll $ 0.01). That is, experts are more manipulable by persuasion than novices.

**Table 5:** Overview of results by experience

	games	N	pers	no pers	binom
	$>$30	17	0.236	0.114	$\top $
	15–30	8	0.344	0.198	$\top $
novice	$<$15	25	0.433	0.312	$\top $
expert	$\geq $15	25	0.274	0.138	$\top $

On the face of it, this is a surprising result. But recall that in a win-lose game, it’s rational to assume, in the absence of information to the contrary, that the persuader’s message isn’t credible (Crawford and Joel Sobel 1982); in other words, the rational default assumption is that the persuasion move isn’t preferable. But this is a *default* assumption: with sufficient resources, one could test the veracity of the persuader’s message, independently of inferences about speaker credibility, via calculations of expected utilities of all the nodes in your (limited) perception of the game tree. Intuitively, novices will be much less likely than experts to carry out such a test with sufficient levels of confidence, however. For example, if the persuasion move is beyond one’s current limited sight on the game, then one would need to add this option, together with its possible subsequent states, to the current game tree, and recompute the expected utilities of all the states in this newly expanded set of possible state transitions.

We don’t have direct and conclusive evidence that novices find such judgements harder to compute than experts. However, we do have indirect and suggestive evidence: the average (Shannon) *entropy* of the build plan(s) that novices choose in the 40 target stimuli, which we estimated via equation (2) (where $\mathit {BP}$ is the set of 4 possible choices of road, settlement, city or card, $n_{a}$ is the number of participants that chose action $a\in \mathit {BP}$ in stimulus *s*, and *N* is the total number of participants), is significantly higher (on average) than those of the experts’ (binomial test, $p = 0.04$);^11^ see Table 7 in the Appendix.

2 $$ \begin{array}{ll} \mathit{entropy}(s) = & {\sum}_{a\in \mathit{BP}} P(a)\ln(P(a))\\ \approx & {\sum}_{a\in \mathit{BP}, n_{a}\neq 0} \frac{n_{a}}{N}\ln(\frac{n_{a}}{N}) \end{array} $$

Thus novices disagree with each other about which build plan is optimal significantly more (on average) than experts do; this in turn suggests that novices find choosing an optimal action harder. That being so, it’s natural to assume that novices also find it harder to *adapt* their judgements about optimal action when attention is drawn to an alternative (perhaps unforeseen) action, and so novices are more often in a situation where they default to assuming the persuader’s message isn’t credible, in which case they aren’t manipulated by it.

The entropy is positive (but non-conclusive) evidence that novices find judgements about optimal action harder than experts, but our experimental set up doesn’t provide any evidence for our conjecture that participants default to assuming the persuader’s message isn’t credible in those states where they have insufficient confidence in their judgements. To provide evidence for this conjecture, one would need to conduct further experiments in which one can control the participants’ perception of the trustworthiness of the persuader. But designing an experiment where one can control, but observe interactions between, speaker credibility vs. inferences about expected utilities in a complex game remains an open research question, and a matter for future work. With that said, note that the initial build plans in Table 7 provide conclusive evidence that novices and experts are significantly different player types, and so one would expect persuasion to have different effects on these two groups. In 47.5% of the target stimuli, the mode for the novices vs. experts is different. For example, *settlement* is the experts’ mode in 23 of the stimuli but it’s the novices’ mode in only 11 stimuli. Conversely, *development card* is the experts’ mode in only 1 stimulus but it’s the novices mode in 12 stimuli. Indeed, their different reactions to persuasion persist across the distinct persuasion types (recall Hypothesis 2), as shown in Table 6. Note in particular that whilst Trade is be the most potent form of persuasion amongst the participants *overall* (see Table 3), it’s extra potent for novices and not significantly potent for experts. Note also that Trade is the only persuasion type whose relative potency differs in one subgroup of participants from its relative potency in the group overall. The reason for its different potency on novices and experts remains an open research question. But it does illustrate how factors that contribute to a participant’s behaviour in complex games is a highly non-trivial task, making it important to acquire data to guide one towards particular hypotheses for further testing.

**Table 6:** The manipulability of novices vs. experts on the distinct types of persuasion move, with a binomial test to measure when the difference in manipulability is significant

argument	novice (< 15 games)			expert (15+ games)		
	pers	no pers	binom	pers	no pers	binom
0$\rightarrow $0	0.220	0.235	p=.65	0.077	0.054	p=.353
0$\rightarrow $1	0.235	0.300	p=.80	0.125	0.000	$\top $
1$\rightarrow $1	0.344	0.276	p=.25	0.189	0.080	p=.256
1$\rightarrow $0	0.389	0.333	p=.39	0.296	0.050	$\top $
x$\rightarrow $y	0.287	0.275	p=.43	0.171	0.056	$\top $
block	0.481	0.493	p=.62	0.509	0.236	$\top $
trade	0.382	0.074	$\top $	0.098	0.056	p=.156
DC/LA	0.667	0.324	$\top $	0.390	0.111	$\top $
rare res	0.688	0.438	$\top $	0.456	0.329	$\top $
Σ	0.433	0.312	$\top $	0.274	0.138	$\top $

The stimuli are all intermediate game states that are far removed from any end states: this is anecessary feature of the experimental design, to ensure the participants have limited sight. But participants don’t play to the end of the game: limited resources prohibit this. So one might be concerned that the participants favour immediately attractive options over those that contribute to winning—indeed, we suggested that gaining immediate VPs may be afactor that influences the potency of apersuasion move. However, there is no evidence that immediate VPs have this effect because participants don’t care about winning the game. If that were the case, then participants would always refuse a $0\rightarrow 1$ offer and always accept a $1\rightarrow 0$ offer; Table 2 shows they didn’t. There is also ample anecdotal evidence from the participants’ comments that their decisions were based on adesire to win, or at least on the relative benefits of outcomes that don’t happen until well into the future. The following are typical examples:

(to justify the declared preferred action): *I would want to free up more settlements for later use*

(commenting on rejecting the persuasion move “… and then you can build asettlement”): *I would have to build the settlement at the wheat port, which isn’t especially useful.*

(on refusing the trade offer): *Since Iwant adevelopment card, the clay wouldn’t really help me get acity afterwards, and if Igot asoldier Icould gain more clay myself anyway.*

(on accepting atrade offer that’s accompanied by the persuasion move “… and then you could buy adevelopment card”): *If Igot asoldier Icould cause problems for the orange and blue players and make progress towards having Largest Army.*

(on refusing the trade offer): *I would want to get the ore from another source, as the orange player is in adangerous position.*

### Discussion

The main purpose of this experiment was to test whether drawing attention to a future move that’s made possible by a proposed (and currently dispreferred) trade increases the likelihood that the human recipient accepts the trade. This main hypothesis was confirmed.

Not all types of persuasions are equally effective, however. Trade has the strongest effect; Blocking the smallest one. Observe that the base likelihood that the recipient accepts a trade offer without the persuasion was *low* for Trade, but *high* for Blocking. We argued that one reason for this difference probably rests with factors that influence whether a particular future option is within the participant’s limited sight, or not. For instance, on the basis of *visual salience*, a Trade persuasion move is more likely than the Blocking move to provide the participant with a new possibility that (until now) were unforeseen (if visual salience correlates with limits on a participant’s perception of their options). But testing whether and how visual salience effects the potency of persuasion in games of limited sight would require further experiments, in which one controls the visual salience of future options. And as we argued in Section 1, even if visual salience is a factor, it is inevitably one of many interacting factors that determine the ways in which a player’s perspective on the game is limited, and it is currently impossible to test a specific model of limited sight via experiments.

Novices and experts differ, with novices accepting more trade offers (with or without persuasion) than experts, but experts being *more manipulable* via persuasion than novices. Our experimental data shows that novices are quite different player types from experts in their initial choices of build plan, and whilst this alone does not explain why novices are less manipulable, it does explain why their manipulability is *different* and also why their likelihood of accepting a trade offer without persuasion is different. We also observed that novices exhibit higher *entropy* in their initial build plan than experts, which suggests that they find the task of computing an optimal action harder. If their confidence in calculating what’s optimal is sufficiently lacking, then it is rational to resort to the default assumption that the persuader’s message isn’t credible (for the experiments are conducted in a win-lose game). So the novices’ higher entropy is positive, but inconclusive, evidence that they are less manipulable than experts because they are more likely to have to rely on the default inference that the message (i.e., that the alternative plan is preferable) isn’t credible.

## General Discussion and Conclusions

Humans face many complex game problems in which they must decide how to act in spite of *limited sight* of the full game (Halpern and Rêgo 2013; Degremont et al. 2016). Currently there is no formally precise framework for predicting human behaviour in such games. Indeed, there is not even any understanding of its required parameters. So what is currently needed is a way of collecting human empirical data on how humans cope with limited sight. This paper aimed to address that gap; we designed experiments in which we attempt to manipulate human participants into changing their plans by presenting them with a persuasion move that draws their attention to a future option that’s different from the one they initially chose.

This human study of persuasion is to our knowledge the first such study that is conducted in a controlled manner but within a domain in which negotiating is only a small part of a very complex game problem. Some of the results of our experiments were surprising. This underlines the need to collect at least some empirical data *before* designing a formal framework for articulating precise hypotheses about human decision making in complex games, which in turn would need to be tested in further experiments.

The experiments reported here do not relate successful persuasion to winning the game—it is impossible to collect sufficient data where humans play a complex game from beginning to end whilst at the same time ensuring that the experimenter can control all relevant factors (our prior work suggests one would need 10K games to get reliable measures, and acquiring 10K human participants is unfeasible). But agent simulations can help fill this gap. The game simulations reported in Guhe and Lascarides (2014a) and Settle (2015) show that if the persuader’s trading strategy, whilst perhaps not optimal (for we don’t know what is optimal), is good enough to ensure that winning the game correlates with more of your trade offers being accepted, then manipulating opponents through persuasion enhances your win rates still further. In conjunction with the human study given here, this will help one build agents that will have competitive performance against humans when negotiating in complex games.

We didn’t address in this paper what potential advantages there might be to letting yourself be persuaded. Given the evidence from the game simulations from Guhe and Lascarides (2014a) and Settle (2015), isn’t it rational simply to disregard all persuasion arguments? Our experimental data doesn’t aim to answer this question; we don’t predict in which *specific* game states (if any) human decisions deviate from what is rational as defined in formal game theory. In fact, we cannot predict this even in principle, because *Settlers* and similar complex games lack any analytic solution. What we have offered instead is an experimental paradigm in which game simulations explore an empirical relationship between persuasion, reaction strategies and winning, whilst the human experiment reported here then validates whether humans react in similar ways, on average, as the agents do in the simulations.

In addition to the factors that we reported on in this paper, many other factors influence whether persuasion has an effect, e.g. players may be less willing to trade with an opponent that they perceive to be manipulative. Players may also engage in (irrational) courses of action simply to take revenge. This will be explored in future work. In future work we also plan to explore other strategies for manipulating the human player’s perspective on the game; e.g., by performing tasks in which the human participants start out unaware of the full rules of the game, and so must learn to adapt their behaviour as and when they discover unforeseen options whilst playing the game.

## Appendix A: Further Factors of the Experimental Design

Because *The Settlers of Catan* is a complex game, there are many more factors that influence a player’s decisions. Below is a list of aspects of the game state that are all possible independent variables for experimentation, but for which we took one of three options: chose a *fixed* value; *ignored* it; or *counterbalanced* it in the stimuli.

- Game map [fixed]: We used the map from *The Settlers of Catan Game Rules and Almanack*.
- Player colour/perspective [counterbalanced]: participants adopted the role of each of the four colours an equal number of times.
- Player turn [fixed]: It was always the participant’s turn.
- Turn phase [fixed]: the dice roll and consequent resource allocation and robber moves were all completed.
- Total number of resources [roughly counterbalanced]: participants never had more than 8 resources; opponents never more than 12.
- Type of piece that can be built before/after the trade [counterbalanced]: Intuitively, there is a bigger incentive to build a settlement/city than a road/development card, because of the increased resource payout.
- Bank/port trade possible [fixed]: The participant lacks resources to trade with the bank or a port (unless she accepts the trade offer).
- Opponents’ hands [ignored]: The opponents’ hand (number of resources, number of cards, number of soldiers, number of pieces left) were largely left as they occurred during the actual games. Adjustments were made for resources (minimum 2, maximum 12) and number of soldiers (for stimuli where the opponent was holding the Largest Army badge).
- Chat/game move history [fixed]: No game history was given.
- Accuracy of beliefs about other players [fixed]: participants had no information about the game history, so they could not form beliefs about other players.
- Number of cards left in the deck [ignored]: Number of cards in the deck was not given.
- Phase of the game [roughly counterbalanced]: The leader had between 2 and 7 VPs.
- Difference in players’ VPs [fixed]: Because we were using simulated games all players were close in terms of VPs. The maximum difference in VPs was usually 2 and never more than 3 VPs. We constrained the VPs for target stimuli for the blocking move in the way we described in Section 5.2.
- Robber location [ignored]: As described above, the robber was in the position as it occurred in the game, never placed on a participant’s hex, with the exception of ‘move robber, get Largest Army’ cases.
- Probability that the board will generate resources for players [ignored]: Players may be more likely to trade if they have a lower probability to obtain resources from the board instead of by trade.

## Appendix B: Instructions

Participants received the following instructions: Please read the following instructions carefully. In this experiment, you will be presented with snapshots of *The Settlers of Catan* games. The games are similar in many respects, because they were played on the standard *Settlers* map by computer agents. Our goal is to compare these agents with expert players, meaning: you. Below is ascreenshot of what you will see in the following, the game snapshot on the left and apanel for your answer on the right. For each situation, we will assign you the role of a specific player that you should adopt (here: the *blue* player). You should assume that you take over the game from the given position and continue to play as this player. In all situations, it is the start of this player’s turn, after the dice have been rolled and the resources have been distributed. Information about the current hand of this player is displayed in the player’s hand panel. This panel contains the following information: In each situation, we will ask you to make two decisions:

1. Assess the given situation and decide what you are planning to build next. Please indicate your choice with one of the radio buttons on the right. (Note: Your opponents won’t know what you say here.)
2. Another player will then make you atrade offer and you have to decide whether you would accept the offer. Again, please indicate your choice with the radio buttons.

Please use the *Comment* field to indicate reasons for your decisions. Although giving comments is optional, we encourage you to write anything that you find worth mentioning about any aspect of your choices or the game position in general. Please comment in particular in those cases when the simple multiple-choice answer does not satisfactorily explain your decision or there are specific strategic reasons for your decision. Examples include:

- You would like to make acounteroffer.
- You are planning to play one of your development cards.
- You think the best plan is to try to get the Largest Army, but you can only indicate that the best next action is to buy adevelopment card.

**Table 7:** The declared preferred build plan of novices (< 15 games) vs. experts (≥ 15 games) in the 40 target stimuli and the entropy of those choices for these two sub-groups of participants, estimated via equation (2)

	Choice of BP for novices (< 15 games)				
Stimulus	Road	Settlement	City	Card	Entropy
1	7	2	10	5	1.26
2	6	6	6	6	1.39
3	17	0	6	1	0.72
4	12	3	8	1	1.11
5	5	4	14	1	1.07
6	9	0	2	13	0.91
7	16	2	4	2	0.98
8	9	8	1	6	1.21
9	17	4	2	1	0.88
10	3	8	4	9	1.29
11	4	14	2	4	1.12
12	4	3	12	5	1.23
13	6	4	5	9	1.34
14	6	4	13	1	1.11
15	14	6	3	1	1.05
16	3	4	6	11	1.26
17	8	2	1	13	1.04
18	6	6	2	10	1.27
19	5	8	4	7	1.35
20	3	3	13	5	1.18
21	4	7	9	4	1.32
22	2	12	1	19	1.05
23	16	6	0	2	0.82
24	4	8	2	10	1.24
25	6	8	4	6	1.36
26	12	4	6	2	1.20
27	7	11	3	3	1.24
28	7	7	2	8	1.29
29	10	10	3	1	1.12
30	11	5	5	3	1.27
31	8	5	5	6	1.37
32	4	7	11	2	1.22
33	6	11	2	5	1.24
34	5	16	2	1	0.94
35	7	13	0	4	0.99
36	4	I5	12	3	1.23
37	8	9	6	1	1.21
38	5	6	3	10	1.30
39	3	8	2	11	1.19
40	4	3	5	12	1.23
avg entropy					1.17
	Choice of BP for experts (15 + games)				
1	4	3	14	4	1.17
2	5	8	9	3	1.31
3	4	11	10	0	1.02
4	9	4	11	1	1.15
5	5	5	15	0	0.95
6	5	14	0	6	0.99
7	8	12	2	2	1.12
8	4	18	1	2	0.86
9	12	8	3	2	1.17
10	4	15	3	3	1.11
11	3	16	5	1	0.99
12	1	6	18	0	0.71
13	4	11	8	2	1.22
14	5	6	13	1	1.13
15	4	13	4	4	1.22
16	4	5	10	6	1.32
17	4	12	3	6	1.24
18	6	5	3	11	1.28
19	7	9	8	1	1.22
20	9	0	15	1	0.80
21	1	6	16	2	0.96
22	1	18	1	5	0.82
23	15	8	2	0	0.87
24	4	17	13	1	0.94
25	2	12	5	6	1.22
26	10	10	3	2	1.19
27	4	16	2	3	1.04
28	12	10	1	2	1.05
29	9	11	3	2	1.19
30	6	13	5	1	1.13
31	3	4	14	4	1.17
32	8	6	9	2	1.28
33	9	16	0	0	0.65
34	3	20	2	0	0.64
35	4	17	3	1	0.94
36	1	8	11	5	1.18
37	2	7	15	1	0.99
38	6	9	5	5	1.35
39	3	13	5	4	1.21
40	2	13	6	4	1.18
avg entropy					1.07

Since there are four possible build plans, the entropy range is [0,1.39]

## Footnotes

1 Note that in in line with standard modal logics of action, $\neg \langle a\rangle \neg \phi $ (“it is not possible that after doing action *a*$\phi $ is false”) is equivalent to $[a]\phi $ (“it is necessarily the case that after doing action *a*$\phi $ is true”).

2 Legal dialogue moves are assumed to be trade offers where the player possesses its giveable resources, and accept, offer or reject in response to a trade offer.

3 For the sake of linguistic economy, we refer to buying a development card as a ‘build’ action. The graphical interface made buying a development card a clear option in the participants’ preference declarations; see Fig. 2 on page 15.

4 For the Build move, the piece mentioned in the move could be road, settlement or city; which one is mentioned depends on the particular target stimulus, via the consistency test described earlier. Similarly, for the Trade move, the type of port or bank and the type of build piece that are mentioned are both determined by the target stimulus, and likewise for the resource that’s mentioned in the Rare Resource move.

5 The piece mentioned could be road, settlement or city, depending on the game state; similarly for the build plan and type of resource that’s mentioned in Trade and Rare Resource persuasion moves.

6 The response times were 1 second on average, compared with an average over the other participants of 85s in the first phase and 35s in the second. Since the experiment is not conducted in the lab, we don’t use reaction times in our analysis.

7 We branched off version 1.1.6 of JSettlers2: http://sourceforge.net/projects/jsettlers2.

8 Available at http://www.catan.com/en/download/?SoC_rv_Rules_091907.pdf

9 Our null hypothesis for the binomial test was that using persuasion does not increase the baseline probability of a trade offer being accepted, which we computed from the cases where no persuasion argument was presented.

10
https://stat.ethz.ch/R-manual/R-devel/library/stats/html/prop.test.html

11 On a null hypothesis where the novices’ entropy is equally likely to be higher or lower than the expert’s entropy.
